# Biomimetic Coating-free Superomniphobicity

**DOI:** 10.1038/s41598-020-64345-1

**Published:** 2020-05-13

**Authors:** Ratul Das, Zain Ahmad, Jamilya Nauruzbayeva, Himanshu Mishra

**Affiliations:** 1 King Abdullah University of Science and Technology (KAUST), Water Desalination and Reuse Center (WDRC), and Biological and Environmental Science and Engineering (BESE) Division, Thuwal, 23955-6900 Saudi Arabia; 2 Present Address: ACWA Power, KAUST ACWA Power Center of Excellence, 4700 King Abdullah University of Science and Technology, Thuwal, 23955 Saudi Arabia

**Keywords:** Surfaces, interfaces and thin films, Surface patterning, Bioinspired materials

## Abstract

Superomniphobic surfaces, which *repel* droplets of  polar and apolar liquids, are used for reducing frictional drag, packaging electronics and foods, and separation processes, among other applications. These surfaces exploit perfluorocarbons that are expensive, vulnurable to physical damage, and have a long persistence in the environment. Thus, new approaches for achieving superomniphobicity from common materials are desirable. In this context, microtextures comprising “mushroom-shaped” doubly reentrant pillars (DRPs) have been shown to repel drops of polar and apolar liquids in air irrespective of the surface make-up. However, it was recently demonstrated that DRPs get instantaneously infiltrated by the same liquids on submersion because while they can robustly prevent liquid imbibition from the top, they are vulnerable to lateral imbibition. Here, we remedy this weakness through bio-inspiration derived from cuticles of *Dicyrtomina ornata*, soil-dwelling bugs, that contain cuboidal secondary granules with mushroom-shaped caps on each face. Towards a proof-of-concept demonstration, we created a perimeter of biomimicking pillars around arrays of DRPs using a two-photon polymerization technique; another variation of this design with a short wall passing below the side caps was investigated. The resulting gas-entrapping microtextured surfaces (GEMS) robustly entrap air on submersion in wetting liquids, while also exhibiting superomniphobicity in air. To our knowledge, this is the first-ever microtexture that confers upon intrinsically wetting materials the ability to simultaneously exhibit superomniphobicity in air and robust entrapment of air on submersion. These findings should advance the rational design of coating-free surfaces that exhibit ultra-repellence (or superomniphobicity) towards liquids.

## Introduction

Liquid-repellent surfaces are utilized in a broad spectrum of applications, such as preventing^1^ and harvesting fog^2^, removing bubbles from aqueous feeds^3^, fluid drag reduction and self-cleaning^4^, preventing adhesion of barnacles onto ship hulls^5^, and anti-corrosion coatings^6^, among others^7^. In this context, superomniphobic surfaces are known to repel polar and apolar liquids, such as water and hexadecane, respectively, and characterized by advancing and receding contact angles satisfying the empirical relations ${\theta }_{{\rm{A}}} > 150^\circ$ and as ${\theta }_{{\rm{A}}}-{\theta }_{{\rm{R}}}\le 10^\circ$^8–10^. Current approaches to realize superomniphobicity necessitate specific chemicals and arbitrary surface roughness or micro/nano patterns. These chemicals induce high interfacial tension at the solid-liquid interface, while the surface roughness facilitates the entrapment of air therein thus lowering the adhesion^11–13^. While there is no *magic* combination of surface chemistry and roughness that guarantees repellence against all known liquids, especially those with high vapor pressure and low surface tensions, the most common choice for the chemicals involves perfluorocarbons, e.g., perfluorooctanoic acid, because of their low affinity towards common polar and apolar liquids^14–16^. However, perfluorocarbons pose environmental concerns^17^, and they are vulnerable to heat, harsh chemicals, organic fouling, and abrasion under turbulent flows. For instance, water repellence of porous anodic alumina (PAA) membranes coated with perfluorodecyltriethoxysilane decreased significantly after 72 hours of immersion in pH 3 water^18,19^, and similar observations have been documented for siloxane coatings and perfluorinated membranes after extended contact with water^20^ or aqueous feeds containing organics^21,22^. Due to the loss of water-repellence, omniphobic surfaces can lose the entrapped air leading to higher frictional drag^23^, and membranes can suffer from pore-filling resulting in process failure^22^. Taken together, the vulnerability of coatings limits their practical applications. Thus, it is desirable to achieve superomniphobicity using common wetting materials, without relying entirely on chemical coatings.

In a landmark paper, Liu & Kim introduced a microtexture that exhibits superomniphobicity regardless of their surface chemistry^24^. Specifically, they microfabricated mushroom-shaped pillars^25,26^, also known as doubly reentrant pillars (DRPs), onto SiO_2_/Si wafers and measured advancing and receding contact angles of a variety of liquids with surface tensions as low as 10 mN/m and found them to be ${\theta }_{{\rm{A}}} > 150^\circ$ and as ${\theta }_{{\rm{A}}}-{\theta }_{{\rm{R}}}\le 10^\circ$. When wetting liquids are placed on DRPs as droplets, the microtexture stabilizes the liquid-vapor interface, trapping air underneath, leading to Cassie-states^27^. The resulting solid-liquid-vapor configuration presents kinetic barrier(s) impeding the transition to the thermodynamically stable Wenzel state;^28^ the barriers can be tuned by the shape and size of the microtexture^13,24^. This is an exciting development because the *functions* of omniphobicity are now achieved by the *structure*, without relying entirely on the surface make-up. In fact, a variety of materials and techniques for manufacturing DRPs have been explored since, including photolithography and dry etching methods for SiO_2_/Si^29–34^, electrical discharge machining (EDM) on steel^35^ and copper^36^, secondary sputtering lithography for polyvinylpyrrolidone^37^, reverse imprint lithography for perfluoropolyether dimethacrylate^38^, 3D printing via two-photon polymerization for acrylic based photoresist^39^, electrically-induced polymer deformation of poly(methyl methacrylate)^40^, and harnessing crack formation/propagation^41^. The expectation is that these approaches would yield greener technologies.

In a recent investigation, it was revealed that even though DRPs demonstrate superomniphobicity towards droplets of wetting liquids in air, the same liquids can spontaneously imbibe into the microtexture on immersion or even if the liquid touches the boundary of the microtexture^42^. Turns out, if a wetting liquid, e.g., water, touches the stems of silica DRPs – at the boundary or due to localized damage – it infiltrates the microtexture due to capillarity, displacing the trapped air. Thus, DRP microtextures are unsuitable for real-world applications that require immersion in liquids, such as those introduced above. It has also been demonstrated that advancing and receding angles^43,44^ could prove to be insufficient/misleading in the assessment of the omniphobicity of surfaces derived from intrinsically wetting surfaces^45^. To remedy this, immersion of surfaces into probe liquids has been advanced as a reliable and simple method for evaluating omniphobicity qualitatively. To overcome the limitations of DRPs, we introduced microtextures comprising (i) doubly reentrant cavities^32,42,46,47^ and (ii) DRPs surrounded by walls^45^ that enable the long-term entrapment of air on immersion in wetting liquids. However, either microtexture does not *repel* liquid drops because of the continuous solid-liquid-vapor triple lines in those architectures, leading to ultralow receding contact angles, ${\theta }_{{\rm{R}}}\approx 0^\circ$^48,49^. Here, through laboratory experiments, we address the fundamental question: “*Is it possible to realize superomniphobic microtextures from wetting materials that enable robust entrapment of air on immersion and repulsion of liquid drops in air?*”.

**Figure 1:**
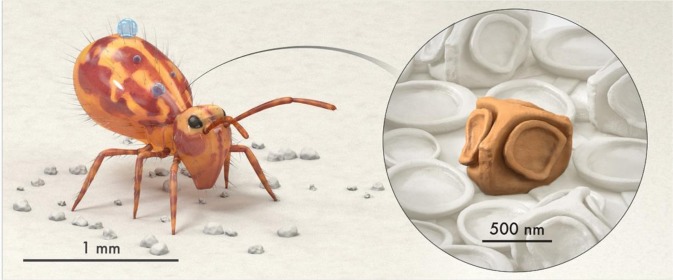
Schematic of *Dicyrtomina ornate* (adapted from ref. ^53^ with permission). Magnified view of the cuticle depicting a cuboidal granule with mushroom-shaped features on each face (artistic reconstruction of a scanning electron micrograph of the cuticle reported in ref. ^51^).

In recent years, a number of surfaces inspired by omniphobic cuticles of springtails (Collembola), soil-dwelling bugs, have been reported^13,24,29–32,35–41,46,50^. Our inspiration came from a report by Werner & co-workers that presented images of cuticles of *Dicyrtomina ornate*, which belong to the order *Symphypleona* and family *Dicyrtomidae* of springtails^51^. Their cuticles are unique even among springtails because they comprise cuboidal secondary granules with a mushroom-shaped feature on each face (Fig. 1). We had no experimental evidence that these granules might contribute to the entrapment of air on accidental submersion in water^52^, but based on our research on gas entrapping microtextured surfaces we surmised that pillars with such a design could prevent lateral imbibition of liquids as well as from the top. However, creating these geometries is a formidable task, for example, by photolithography and dry etching. Herein, we employ a two-photon polymerization technique to realize arrays of DRPs surrounded by a boundary of *Dicyrtomina ornata*-inspired pillars that have mushroom-shaped caps on top and laterally. Variations of this design are also presented towards a proof-of-concept demonstration of microtextured surfaces, derived from intrinsically wetting materials that can entrap air robustly on immersion and exhibit superomniphobicity against liquid droplets in air. Hereafter, we refer to these gas entrapping microtextured surfaces by the acronym GEMS.

## Results and Discussion

### Omniphobicity in air

**Figure 2:**
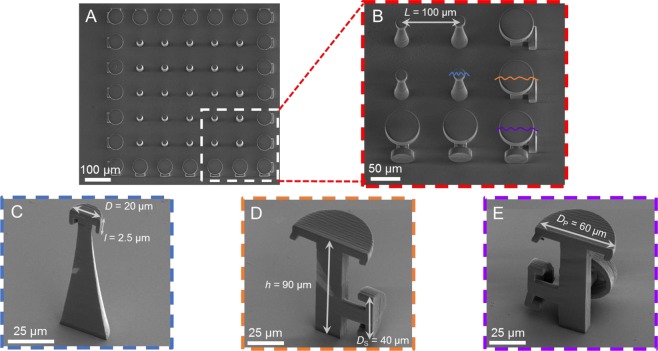
(**A**) Scanning electron micrographs of arrays of 3D printed doubly reentrant pillars (DRPs) surrounded by *Dicyrtomina ornate-inspired* DRPs (DO-DRPs). (**B**) Magnified view of the bottom right corner of the image (**A**). (**C–E**) Cross-sections of DRP, DO-DRP with one lateral cap, and DO-DRP at the corners with two lateral-caps.

Using a two-photon polymerization platform, GEMS comprising arrays of DRPs surrounded by *Dicyrtomina ornate*-inspired DRP’s (DO-DRPs) were realized using a methacrylate based negative tone photoresist (IPS, Nanoscribe GmbH) on SiO_2_/Si wafers (Fig. 2, Methods). The diameter of the mushroom-shaped caps of the DRPs was *D* = 20 µm and the center-to-center distance between the pillars (the pitch) was *L* = 100 µm. Surrounding the DRP array were the DO-DRPs, each with a mushroom-shaped cap (on top) of diameter, *D*_P_ = 60 µm and a lateral mushroom-shaped cap of diameter, *D*_S_ = 40 µm. The DO-DRPs at the corners had two orthogonally positioned lateral caps of diameter *D*_S_. The pitch and height of all the pillars were, respectively, *L* and *h* = 90 µm. Water, hexadecane, and isopropanol were the probe liquids, and their apparent contact angles on flat and homogeneous IPS surfaces in air were, respectively, *θ*_r_ = 70° ± 2°, *θ*_r_ ≈ 10° ± 2°, and *θ*_r_ ≈ 9° ± 3° (we consider these angles to be the actual contact angles (*θ*_o_) for our theoretical analysis (SI, Sections S1, S2). For the terminology, please see ref. ^10^). Next, advancing (${\theta }_{{\rm{A}}}$) and receding (${\theta }_{{\rm{R}}}$) contact angles were measured on the GEMS by dispensing and retracting the probe liquids at 0.2 µL/s. (Methods, Tables 1 and 2)^43,44^. The apparent advancing contact angles on GEMS were *θ*_A_> 150°; the liquid-solid work of adhesion was minimal as evidenced by the bouncing off of droplets dropped from a height of *h* ≈ 3 mm and contact angle hysteresis, $\Delta \theta ={\theta }_{{\rm{A}}}-{\theta }_{{\rm{R}}}\approx {30}^{^\circ }$ (Table 2, Movies S1-2)^54,55^. The sizes of the liquid droplets in these experiments were below their respective capillary lengths, given by the formula^11^:

$${\lambda }_{c}=\sqrt{\frac{{\gamma }_{{\rm{LV}}}}{\rho g}}$$

where, *γ*_LV_ is the surface tension, *ρ* is the density, and *g* is the acceleration due to gravity. Furthermore, since the surface roughness of the GEMS was much smaller than the volume of the drops of the probe liquids, we could apply the Cassie-Baxter model to gain insights into the wetting behavior of GEMS^13,27,56^. In fact, we found a reasonable agreement between the predicted and the observed apparent advancing contact angles on GEMS (Sections S1-2).

**Table 1:** Physical properties of probe liquids, at 293 K and 1 atm^57–61^ and their contact angles on smooth and homogeneous IPS (methacrylate based negative tone photoresist, Nanoscribe GmbH).

Probe liquids	Surface tension, ${{\boldsymbol{\gamma }}}_{{\bf{L}}{\bf{V}}}$ (mN.m^−1^)	Density, *ρ* (kg.m^−3^)	Vapor pressure, *p*_v_ (Pa)	Capillary length, λ_c_ (mm)	Contact angles on flat IPS in air		
					Actual or intrinsic (${{\boldsymbol{\theta }}}_{{\bf{o}}}$)	Advancing (${{\boldsymbol{\theta }}}_{{\bf{A}}}$)	Receding (${{\boldsymbol{\theta }}}_{{\bf{R}}}$)
Water	72	997	2300	2.71	70° ± 2°	79° ± 2°	27° ± 2°
Hexadecane	28	773	10	1.92	10° ± 2°	12° ± 2°	0°
Isopropanol	23	786	4400	1.73	9° ± 3°	11° ± 2°	0°

**Table 2:** Contact angles – actual (or intrinsic, ${{\boldsymbol{\theta }}}_{{\bf{o}}}$), apparent advancing (${{\boldsymbol{\theta }}}_{{\bf{R}}}$) and receding (${{\boldsymbol{\theta }}}_{{\bf{A}}}$) - of droplets of water, hexadecane, and isopropanol on the GEMS presented in Fig. 2.

Contact angles	Probe liquids		
	Water	Hexadecane	Isopropanol
${{\boldsymbol{\theta }}}_{{\bf{o}}}$	70° ± 2°	10° ± 2°	9° ± 3°
${{\boldsymbol{\theta }}}_{{\bf{R}}}$	172° ± 2°	168° ± 3°	164° ± 4°
${{\boldsymbol{\theta }}}_{{\bf{A}}}$	143° ± 2°	140° ± 3°	137° ± 3°

### Omniphobicity under immersion

**Figure 3:**
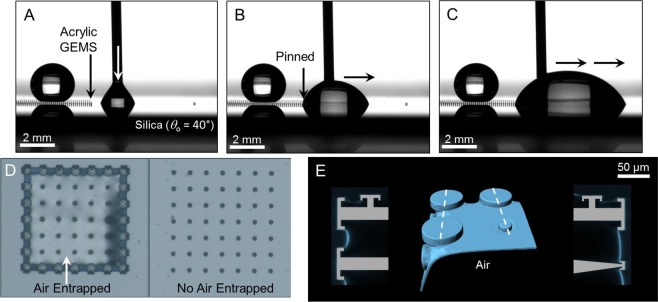
Intrusion from boundary: (**A–C**) Water droplets placed at the boundary of acrylic GEMS (*θ*_o_ = 70° ± 2°) do not infiltrate the microtexture, unlike in the case of DRPs as controls. GEMS under immersion: (**D**) Optical images (top-view) of GEMS comprising DRPs with a lining of DO-DRPs (left) and without (right). Consequences on the entrapment of air inside the GEMS underwater: (left) water did not imbibe, and (right) water imbibed spontaneously. (**E**) Computer-enhanced 3D reconstructions of the air-water interface in contact with GEMS after 10 min of immersion. The image shows the top-left corner of the left-panel in (**D**) where the water menisci are stabilized by the microtexture; the blue (false) color represents the air-water interface while the grey color represents acrylic pillars. Cross-sectional views along the white dashed lines are shown on either side.

Next, we investigated the consequences of the probe liquids touching the boundary of GEMS. In contrast to the acrylic DRPs that got instantaneously infiltrated by water (*θ*_o_ = 70° ± 2°) (Movie S3; see Fig. S2 for silica DRPs), acrylic GEMS prevented lateral imbibition (Fig. 3A–C and Movie S4). In fact, when an advancing water drop approaches the boundary comprising DO-DRPs, it is repelled (Fig. 3A–C). Next, the effects of immersion were investigated by placing GEMS in petri dishes and introducing water at a rate of 1 mL/min to realize a height of *z* ≈ 5 mm. In this scenario, GEMS entrapped air robustly underwater, maintaining Cassie-states, while DRPs got filled instantaneously (*t* < 0.1 s) (Fig. 3D). The isometric reconstruction of the GEMS-air-water interface through confocal laser scanning microscopy revealed that the DO-DRPs stabilized the laterally-intruding water meniscus (Fig. 3E, Section S3). Specifically, the curvature or capillary pressure preventing the imbibition is given by the formula^62^, ${P}_{{\rm{L}}}={\gamma }_{{\rm{LV}}}\times (1/{R}_{1}+1/{R}_{2})\cos \,{\theta }_{{\rm{o}}}$, where ${\gamma }_{{\rm{LV}}}$ is the liquid-vapor interfacial tension, *θ*_o_ is the intrinsic (or actual) contact angle of water droplets on a smooth and homogeneous acrylic surface, and *R*_1_, and *R*_2_ are the radii of curvatures of the air-water interface stabilized at mushroom-shaped caps. It should be realized that these metastable Cassie-states would transition to the fully-filled (or the Wenzel) state over time^56^. In fact, the entrapped air in the microtexture was gradually lost in 96 hrs (4 days) due to the capillary condensation of water that also expedited the dissolution of entrapped gas in water^46^. More experimental observations on the role of humidity on wetting transitions in GEMS are presented later.

**Figure 4:**
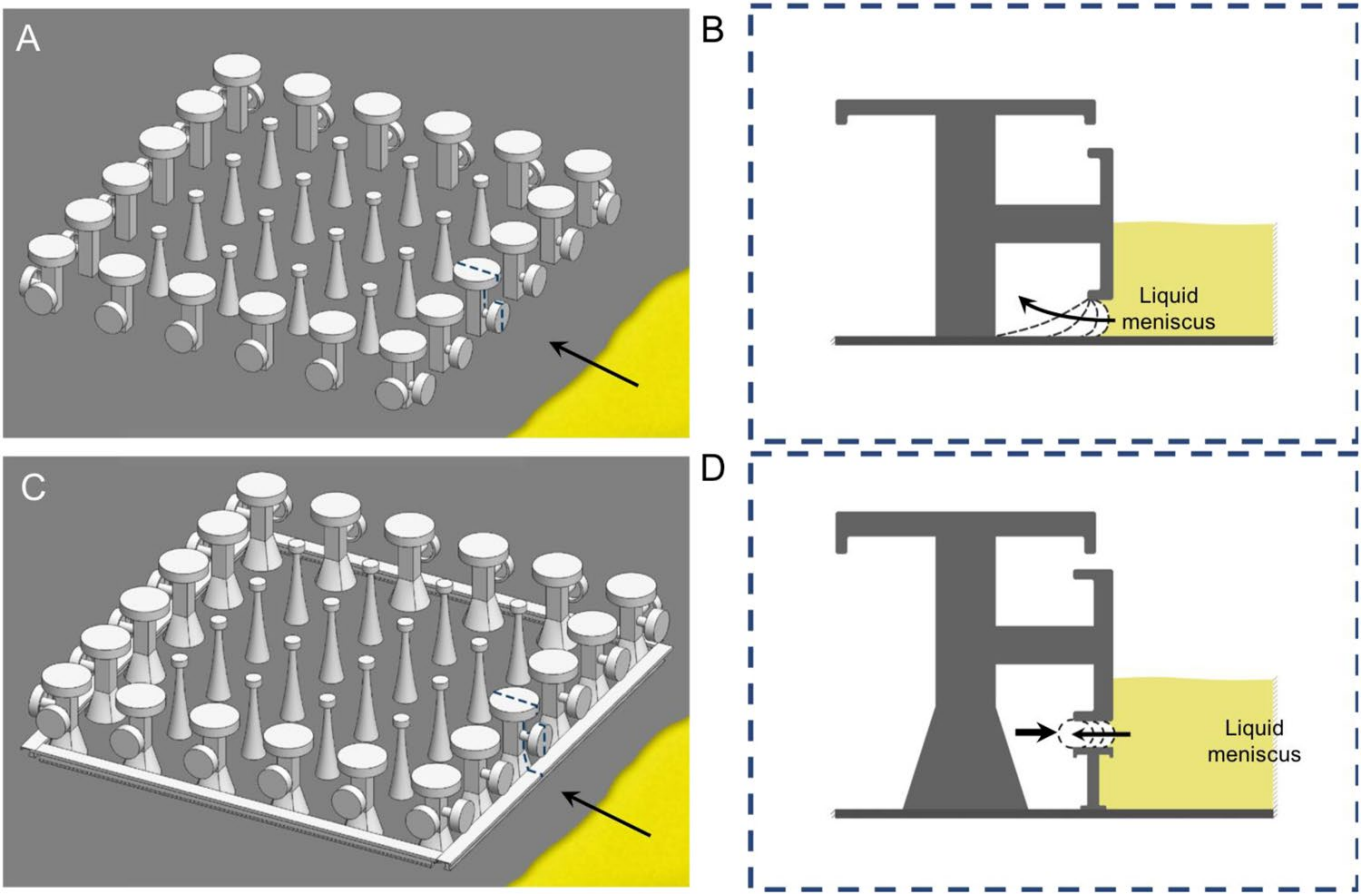
Conceptual schematic of the immersion of DRPs surrounded by a row of DO-DRPs under a highly wetting liquid (*θ*_o_ ≈ 10°). In this case, with the stems of pillars spontaneously imbibe the liquid, which then displaces the trapped air and fills through. (**A**) Liquid advancing towards GEMS; (**B**) Zoomed-in view of the cross-section along the dotted line in (**A**) at the time the liquid touches the boundary pillars. Due to its wetting nature, the liquid advances through the space between the lateral cap and the floor and reaches the pillar stem and invades. To avoid this, we built a wall with a doubly reentrant profile underneath the lateral cap. (**C**) Modified-GEMS with a perimeter of a short wall with a doubly reentrant profile (on top) that passed right below the lateral-caps of the boundary DO-DRPs; **(D)** Zoomed-in view of the cross-section along the dotted line in (**C**). The modified-GEMS presents an additional reentrant (turning) point to prevent the laterally invading liquid (showed by dotted curved lines and arrows).

Next, we subjected GEMS to liquids of lower surface tensions: hexadecane and isopropanol were used as probe liquids. GEMS were placed in petri dishes and liquids were introduced at the rate of 1 mL/min to achieve a column of height *z* ≈ 5 mm. In this case, both the liquids imbibed spontaneously into the microtexture, pushing out the trapped air. This outcome was due to the ultralow apparent angles of hexadecane (or isopropanol) on the smooth IPS surface (*θ*_r_ ≈ 10° ± 2°; Table 1) – the liquid meniscus reached the stems of the pillars laterally underneath the caps and intruded further (Fig. 4A,B). In response, we iterated the GEMS design, by reducing the gap between the side-caps and the floor, but that did not prevent the outcome either (Fig. S3, Movie S5). So, we introduced a perimeter of a short wall with a doubly reentrant profile (on top) that passed right below the lateral-caps of the boundary DO-DRPs **(**Fig. 4C,D**)**. We expected that these modified-GEMS would present an additional reentrant (turning) point to prevent the laterally invading liquid (notice the dotted lines in Fig. 4D), which GEMS do not offer (Fig. 4A,B).

**Figure 5:**
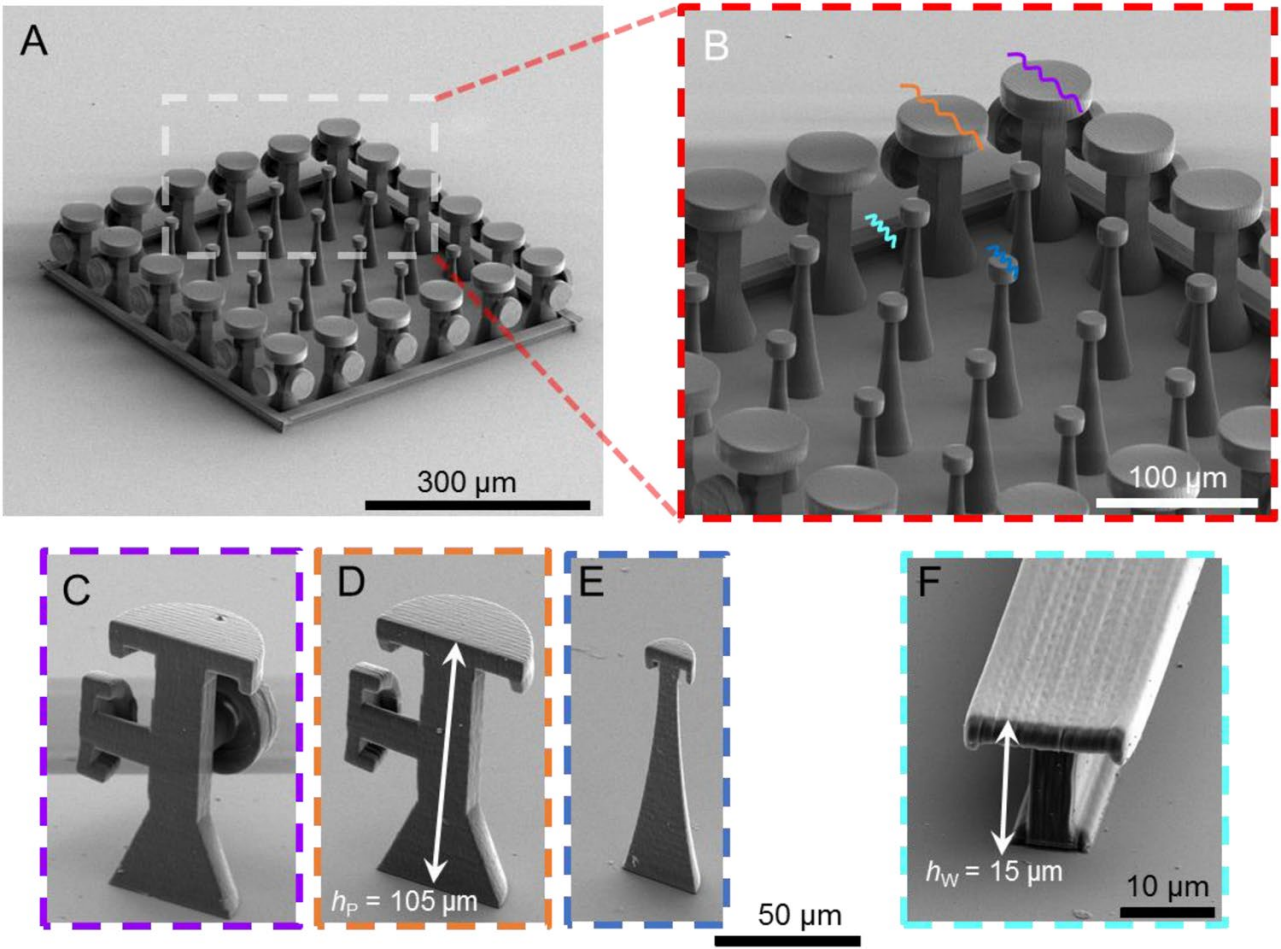
Scanning electron micrographs of arrays of GEMS design comprising an array of DRPs lined with DO-DRPs and a short wall with doubly reentrant profile. (**A,B**) Shows the array with all the key design features, (**C–E**) shows the cross-sections of the pillars. (**F**) The cross-section of the doubly reentrant wall. *h* represents the height of the pillars and wall respectively.

Specifically, we reinforced the boundary of GEMS with the short wall of height *h*_w_ = 15 µm using the two-photon polymerization technique; the height of all the pillars were increased to *h*_P_ = 105 µm (Fig. 5).

**Figure 6:**
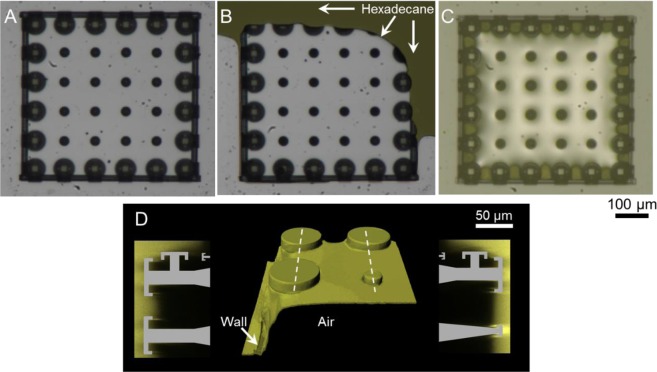
When the actual (or intrinsic) contact angle of hexadecane on acrylic is increased to *θ*_o_ ≈ 70° by FDTS coating, the modified-GEMS entrap air on immersion in hexadecane. (**A**) Top-view of GEMS in air, comprising an array of DRPs surrounded by a row of DO-DRPs and a short wall underneath the side caps. (**B**) Hexadecane is introduced. (**C**) Air is entrapped inside the microtexture immersed under a hexadecane column of height *z* ≈ 5 mm. (**D**) Computer-enhanced 3D reconstruction of the hexadecane-air interfaces at the top left corner of the sample after 10 min. The yellow color corresponds to the interface of hexadecane and grey shows the acrylic pillars. Cross-sectional views along the white (dashed) lines are shown on either side.

The resulting modified-GEMS exhibited similar advancing/receding angles with the probe liquids as the previous one, i.e. without the wall, as the liquid drops never touched the wall during contact angle measurements. The presence of the short wall fortified the design and increased the stability of the pinned liquid meniscus; the modified-GEMS entrapped air underwater for over 2 weeks (Fig. S4). On immersion in hexadecane, the GEMS could entrap air inside them. However, it was not a robust arrangement - mechanical perturbations would lead to liquid intrusion (Movie S6). In fact, it has been demonstrated previously that mechanical agitation/vibrations could destabilize metastable Cassie-states due to the de-pinning of the contact line, leading to wetting transitions and partial/complete loss of the entrapped air^63^. We submit that this GEMS approach can entrap air robustly under liquids that cast intrinsic contact angles ${\theta }_{{\rm{o}}}$> 40°, such that the pinning forces are sufficient, this strategy is vulnerable if the surface is extremely liquid-loving. e.g., ${\theta }_{{\rm{o}}} < {10}^{^\circ }$. In the latter scenario, the pinning force, ${F}_{{\rm{Pin}}}\propto {\gamma }_{{\rm{LV}}}\times (\cos \,{\theta }_{{\rm{R}}}-\,\cos \,{\theta }_{{\rm{A}}})\approx 0$, where ${\gamma }_{{\rm{LV}}}$ is the liquid-vapor interfacial tension and ${\theta }_{{\rm{A}}}$ and ${\theta }_{{\rm{R}}}$ are near-equal receding and advancing angles, respectively. To increase the interfacial tension at the solid-liquid interface and ${\theta }_{{\rm{o}}}$, we coated acrylic GEMS with perfluorodecyltrichlorosilane (FDTS) by following the protocol described in ref. ^64^ (Methods, Table 3). The advancing and receding contact angles for hexadecane thus increased to *θ*_A_ ≈ 79° and *θ*_R_ ≈ 25°, and the pinning force also increased (Table 3). The modified-GEMS could now robustly entrap air under immersion in hexadecane (Fig. 6A–D) for over 336 hrs (or 2 weeks), after which the experiment was discontinued. In contrast, FDTS-coated GEMS (without the wall) still could not entrap air. We consider that the wall and the side-cap presented an additional reentrant (turning) point to prevent the laterally invading liquid (notice the dotted lines in Fig. 4D and compare with Fig. 4B). Crucially, the wall also enhanced the pinning force, ${F}_{{\rm{Pin}}}=L\times {\gamma }_{{\rm{LV}}}\times (\cos \,{\theta }_{{\rm{R}}}-\,\cos \,{\theta }_{{\rm{A}}}),$ because it contributed to a significantly longer solid-liquid-vapor (triple) interface length, *L*, in comparison to GEMS, which only has a discontinuous triple line at the lateral pillars. Movie S7 juxtaposes these starkly different performances of FDTS-coated GEMS with and without walls under immersion in hexadecane, thus underscoring the role of this design.

**Table 3:** Contact angles – actual (or intrinsic, ${\theta }_{{\rm{o}}}$), static (${\theta }_{{\rm{r}}}$), advancing (${\theta }_{{\rm{A}}}$), receding (${\theta }_{{\rm{R}}}$) – and pinning force, ${F}_{{\rm{Pin}}}\propto {\gamma }_{{\rm{LV}}}\times$ ($\cos \,{\theta }_{{\rm{R}}}-\,\cos \,{\theta }_{{\rm{A}}})$, of droplets of water, hexadecane, and isopropanol on bare and FDTS coated flat IPS (methacrylate based negative tone photoresist, Nanoscribe GmbH).

Probe liquids	Contact angles on FDTS-coated, flat IPS surface			${{\boldsymbol{F}}}_{{\bf{P}}{\bf{i}}{\bf{n}}}\propto {{\boldsymbol{\gamma }}}_{{\bf{L}}{\bf{V}}}\times$ (${\bf{c}}{\bf{o}}{\bf{s}}{{\boldsymbol{\theta }}}_{{\bf{R}}}-{\bf{c}}{\bf{o}}{\bf{s}}{{\boldsymbol{\theta }}}_{{\bf{A}}})$	
				Bare IPS	FDTS-coated IPS
	Actual (or intrinsic, ${{\boldsymbol{\theta }}}_{{\bf{o}}}$)	Advancing (${{\boldsymbol{\theta }}}_{{\bf{A}}}$)	Receding (${{\boldsymbol{\theta }}}_{{\bf{R}}}$)		
Water	106° ± 1°	111° ± 2°	47° ± 2°	50.4	74.9
Hexadecane	70° ± 2°	79° ± 2°	25° ± 2°	0.6	20.0
Isopropanol	35° ± 2°	37° ± 2°	0°	0.4	4.6

In the case of isopropanol, the FDTS-coating did not increase the intrinsic contact angle significantly, as has been noted by others^65^. Thus, the pinning forces, ${F}_{{\rm{Pin}}}\propto {\gamma }_{{\rm{LV}}}\times (\cos \,{\theta }_{{\rm{R}}}-\,\cos \,{\theta }_{{\rm{A}}})$ did not increase much (Table 3) and the modified-GEMS failed to robustly entrap air on immersion; additional factors contributing to the failure included isopropanol’s low surface tension, high vapor pressure, and capillary condensation inside the microtexture (Fig. S5, Movie S8). Thus, we are inclined to say that it remains a challenge to entrap air under such liquids using GEMS or with coating-based approaches. Next, we evaluate the effects of capillary condensation of water onto GEMS.

**Figure 7:**
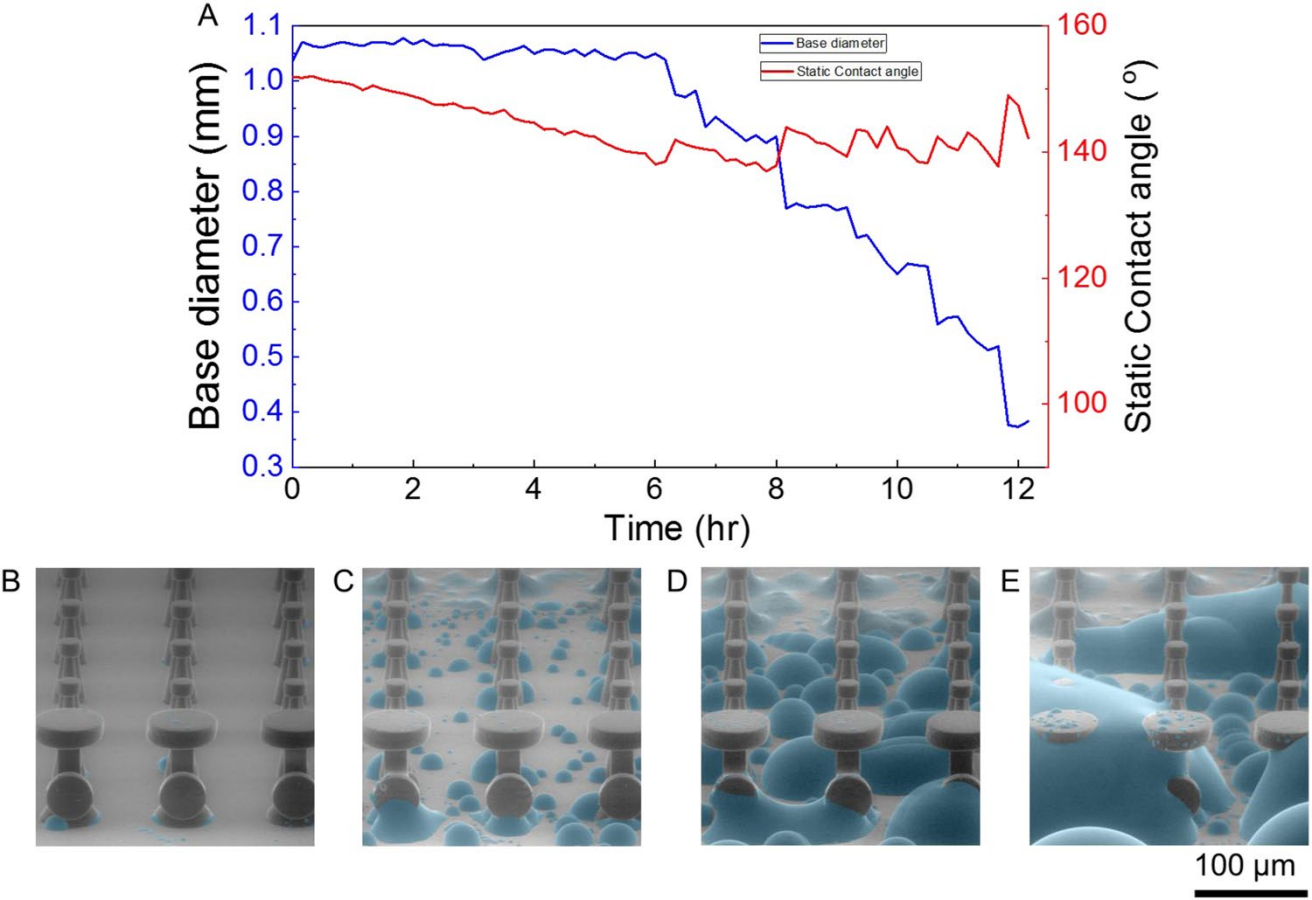
(**A**) Time-dependent contact angles and base diameters of a drop of water placed onto GEMS in an environment with a relative humidity of 92% ± 1%. (**B–E**) Environmental scanning electron microscopy images of GEMS showing the formation of condensate in the microstructure due to capillary condensation.

The capillary condensation of liquids inside GEMS can compromise their ability to robustly entrap air. GEMS can robustly *repel* drops of IPA placed on them because the pillars-based microtexture facilitates the diffusion of the IPA vapor from beneath the drops into the atmosphere (Fig. S6). For instance, we investigated the time-dependence of the static contact angles and base diameters of 6 µL water drops placed onto GEMS under 92% relative humidity. These slowly evaporating drops did not penetrate into the GEMS for ~12 hrs during which they lost about 95% of their volume. (Figs. 7A, S7). On immersion, however, the vapor cannot escape directly to the atmosphere and their capillary condensation depends on the liquid vapor pressure and surface wettability and topography. To observe how capillary condensation might drive wetting transitions in our GEMS, we utilized environmental scanning electron microscopy (Methods, Fig. 7B–E). Below the dew-point of water, we observed drop-wise condensation at the base; water droplets merged and grew with time to reach the top of the pillars, which would drive wetting transitions. To combat wetting transitions due to intense capillary condensation, chemical make-up and design changes would be needed to, respectively, delay and remove the condensed liquid to restore the entrapped air.

**Figure 8:**
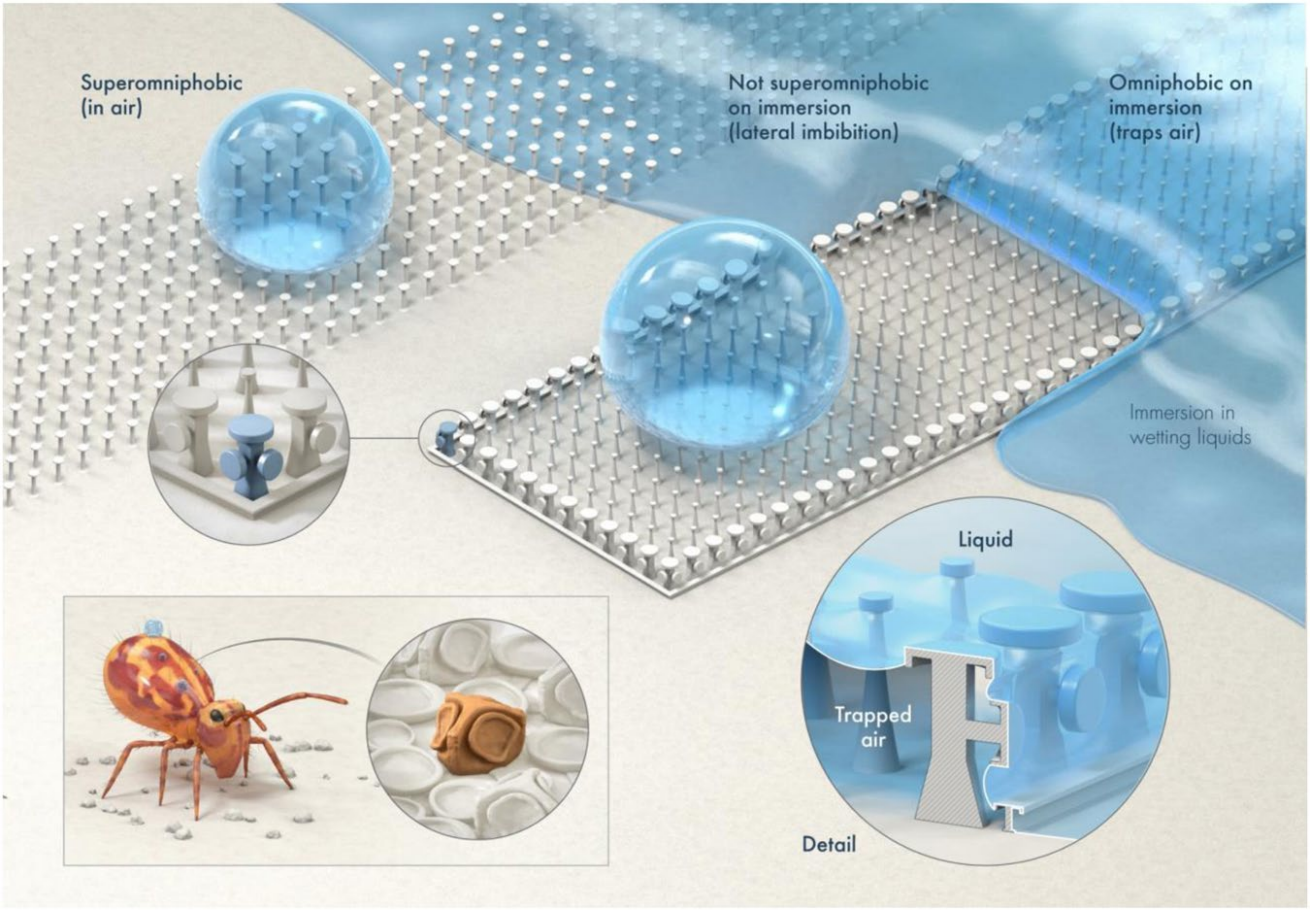
A summary of this work. Inspired by the microstructure of secondary granules in the cuticles of *Dicyrtomina ornate*^51^, we investigated how DO-DRPs might contribute towards preventing the lateral imbibition of wetting liquids into DRP arrays. As shown on top, arrays of coating-free DRPs exhibit superomniphobicity in terms of contact angles, but wetting liquids, e.g., water and hexadecane, instantaneously infiltrate laterally into them, limiting their practical use. To address this, we created a perimeter of DO-DRPs and a short wall around DRP arrays. The resulting microtexture robustly prevented the lateral imbibition of water and hexadecane, while also exhibiting superomniphobicity in air. Thus, this work is a step towards realizing robust, coating-free superomniphobicity – in air and on submersion.

We hope that this time-dependent assessment of GEMS architectures with multiple probe liquids has semi-quantitatively revealed the strengths and weaknesses of this approach. While we have suggested the lower-bound for liquid contact angles for GEMS to be ${\theta }_{{\rm{o}}}$> 40°, surface engineers should consider the specifics of the microtexture, pinning forces, liquid vapor pressure, and target applications to outline their *structure-function* landscape. Figure 8 depicts the conceptual advance presented in this work – GEMS comprising DRPs surrounded by DO-DRPs exhibit superomniphobicity in air and also robustly entrap air on immersion.

## Conclusion

To our knowledge, this is the first-ever demonstration of a microtexture derived from an intrinsically wetting material that not only exhibits superomniphobicity in terms of contact angles but also robustly entraps air upon immersion. Our inspiration for these gas-entrapping microtextured surfaces (GEMS) came from *Dicyrtomina ornate*, whose secondary cuticular granules have cuboids with mushroom-shaped features/caps on each face. We realized that such a design could prevent the lateral imbibition of wetting liquids into any microtexture. To realize the complex bio-inspired geometry, a two-photon polymerization platform was utilized. Additionally, we introduced a perimeter of a short wall with a doubly reentrant profile (on top) that passed right below the lateral-caps of the boundary DO-DRPs that presented additional reentrant bottlenecks against the lateral invasion of liquids. Using water, hexadecane, and isopropanol as the probe liquids, we investigated the strengths and weaknesses of these microtextures. We found that the GEMS-approach is reliable when the actual (or intrinsic) contact angles are not too low, e.g., ${\theta }_{{\rm{o}}} > {40}^{^\circ }$. For example, in the context of water, immersion-proof superhydrophobicity can  be achievable following this approach using common plastics, e.g., polyvinyl alcohol (${\theta }_{{\rm{o}}}\approx {51}^{^\circ }$), poly(methyl methacrylate) (PMMA), and poly (ethylene terephthalate) (${\theta }_{{\rm{o}}}\approx {72}^{^\circ }$) that are also significantly less expensive than perfluorocarbons^57,66^. However, for wetting liquids with high vapor pressures, GEMS might not robustly entrap air on immersion. Thus, new design strategies for delaying the capillary condensation, removing the condensate, and replenishing air should be explored^67–69^. Furthermore, we were unable to assess the mechanical durability of GEMS, for instance, under a running stream of water^20^, because of the mechanical fragility of samples prepared by this microfabrication platform. While two-photon polymerization is not a scalable platform at this point, its use enabled us to realize the proof-of-concept demonstration; further research is needed to explore the *structure*-*function* landscape and translate the design principles using scalable techniques. For instance, a case-by-case time-dependent assessment of GEMS structural features, intrinsic contact angles, contact angle hysteresis, and liquid vapor pressure would inform on the applicability of this approach for a given liquid-solid-vapor system. Applications abound – for instance, the recent proof-of-concept demonstration of the first-ever (coating-free) membranes derived from low-cost hydrophilic materials for water desalination by membrane distillation underscores the promise of this approach^50,70^. Thus, we hope that this report heralds concerted research into GEMS towards realizing greener and sustainable technologies for separation and purification and reducing frictional drag, among others, through advances in additive manufacturing^71^ and laser micromachining^72^.

## Methods

### Microfabrication

Biomimetic microstructures were fabricated by a two-photon polymerization technique (2PP) that utilizes a methacrylate based negative tone photoresist IPS (Nanoscribe GmbH)^73–76^. Silicon wafers (p-doped, 4” diameter and 300 µm thick) afforded smooth surfaces for the microtextures. To this end, Si wafers were cut into 15 mm × 15 mm pieces, rinsed with isopropyl alcohol, and blown dry with nitrogen gas. This was followed by spin-coating an ultrathin layer of adhesion promoter VM-651 (0.1% v/v solution of VM-651 in deionized water, spin-coated at 3000 rpm for 60 seconds), which improves the adhesion of the photoresist with the wafer^77^. The microstructures were fabricated using a commercial workstation (Photonic Professional GT, Nanoscribe GmbH) that exploits a two-photon absorption polymerization process. The setup is equipped with a 780 nm femtosecond laser that delivers 100 fs pulse duration with an 80 MHz repetition rate and 50 mW laser power to achieve a lateral resolution of less than 100 nm and a scan speed greater than 30000 µm-s^−1^. We used a 25X objective (0.8 NA, Zeiss, Plan Apochromat). A stereolithography file (.stl) of a single microstructure was created using the *Solidworks* software. The design was then sliced with the help of the *DeScribe* software in a layer-by-layer format, which converts the desired structures to a specialized General Writing Language (GWL) to generate three-dimensional structures. *DeScribe* was also used to combine different microstructures together to form an array. The laser power and the scan speed were set at 35% (AOM = 1.10, PowerScaling = 1.10) and 10000 µm-s^−1^. The vertical scan was controlled by a high precision piezoelectric stage and the horizontal scan was controlled by a galvanometer (GalvoScan mode). The slicing and hatching distances were set at 0.5 µm and 200 nm for all the microstructures. After printing, the silicon substrate with the photoresist was immersed in mr-Dev 600 (Micro Resist Technology GmbH, Germany) for ~20 min to develop and remove the unexposed photoresist. This was followed by immersion in isopropyl alcohol (~10 min) to dissolve any excess photoresist and developer left behind. The samples were then dried in a vacuum oven for 24 hours at 50 °C to remove any excess solvent. Typical samples had an array of DRPs surrounded by DO-DRPs, which had one or two lateral caps depending on whether they were in a row or at the corner.

### Characterization

A Kruss Drop Shape Analyzer DSA100 was used to determine the static, advancing/receding contact angles with deionized water, hexadecane and isopropanol (IPA) at a rate of 0.2 μL-s^−1^. A 2 μL water drop was placed on the surface to determine the static contact angle, the drop later was inflated at a rate of 0.2 μL-s^−1^, till it reached a volume of 10 μL to measure the advancing/receding angles. A tangential fit was used to determine the contact angles from the droplet image, using the *Advance* software. Samples were coated with a 3 nm Ir layer prior to scanning electron microscopy (Quanta 3D). For immersion studies, samples were immersed under a 5 mm high probe liquid column and imaged with an optical microscope. Immersion under hexadecane was filmed using Edgetronic high-speed camera with a Qioptics objective (focal length - 9.5 cm) at 3000 fps. A Phantom v1212 high-speed camera from Vision Research was used to record liquid drops impacting the surfaces at 10000 fps. A Zeiss LSM710 upright confocal microscope was used to obtain a computer-enhanced 3D reconstruction of liquid-vapor interfaces on immersion in water and hexadecane using Rhodamine B and Nile Red fluorescent dyes respectively (Section S3).

### FDTS coating

The dry samples were activated for 30 min in a Diener Electronics O_2_ plasma system (Atto model) at 100% power (200 W) using O_2_ gas (99.9% purity) with a flow 16.5 sccm, the chamber was maintained at 0.3 mbar pressure. The samples were then transferred to a Molecular Vapor Deposition system (MVD) (Applied Microstructures MVD100E) for FDTS (Perfluorodecyltrichlorosilane) coating. The FDTS pressure was set at 0.5 mTorr and the water pressure was 6 mTorr, after injection into the chamber the reaction was carried for 15 min. This entire cycle was repeated thrice, after which the samples were transferred to a vacuum oven at 50 °C for 3 hours to remove any physisorbed FDTS. FDTS coated samples were used for immersion testing using hexadecane.

### Environmental scanning electron microscopy (EnSEM)

Quanta 600 SEM was used to observe condensation on our GEMs. Sample was maintained at temperature, T ≈ 2 °C. By varying the chamber pressure (P ≈ 750–810 Pa) the relative humidity was stabilized at 100%. The images were taken using secondary electron detector at 10 kV accelerating voltage, beam current 0.45 nA and a working distance of 4–5 mm.

## Supplementary information

Supplementary Movie S2

Supplementary Movie S3

Supplementary Movie S4

Supplementary Movie S5

Supplementary Movie S6

Supplementary Movie S7

Supplementary Movie S8

Supplementary Information.

Supplementary Movie S1

## Data Availability

All data needed to evaluate the conclusions in the paper are present in the paper and/or the Supplementary Materials. Additional data related to this paper may be requested from the authors.
