# Supplementary material for: Biomimetic Coating-free Superomniphobicity
Source: Sci Rep. 2020 May 13;10:7934. doi: 10.1038/s41598-020-64345-1 (PMC7221082; doi:10.1038/s41598-020-64345-1)
Supplement: Supplementary file 8 — Supplementary Information. [file 41598_2020_64345_MOESM8_ESM.docx]

Supporting Information

**Biomimetic Coating-free Superomniphobicity**

Ratul Das, Zain Ahmad, Jamilya Nauruzbayeva, Himanshu Mishra^*^

King Abdullah University of Science and Technology (KAUST), Water Desalination and Reuse Center (WDRC), and Biological and Environmental Science and Engineering (BESE) Division, Thuwal 23955-6900, Saudi Arabia

^*^Corresponding author: [himanshu.mishra@kaust.edu.sa](mailto:himanshu.mishra@kaust.edu.sa)

**Section S1**: Calculation of liquid-solid (*φ*_LS_) and liquid-vapor (*φ*_LV_) area fractions when a liquid drop is placed on the microtextured surface


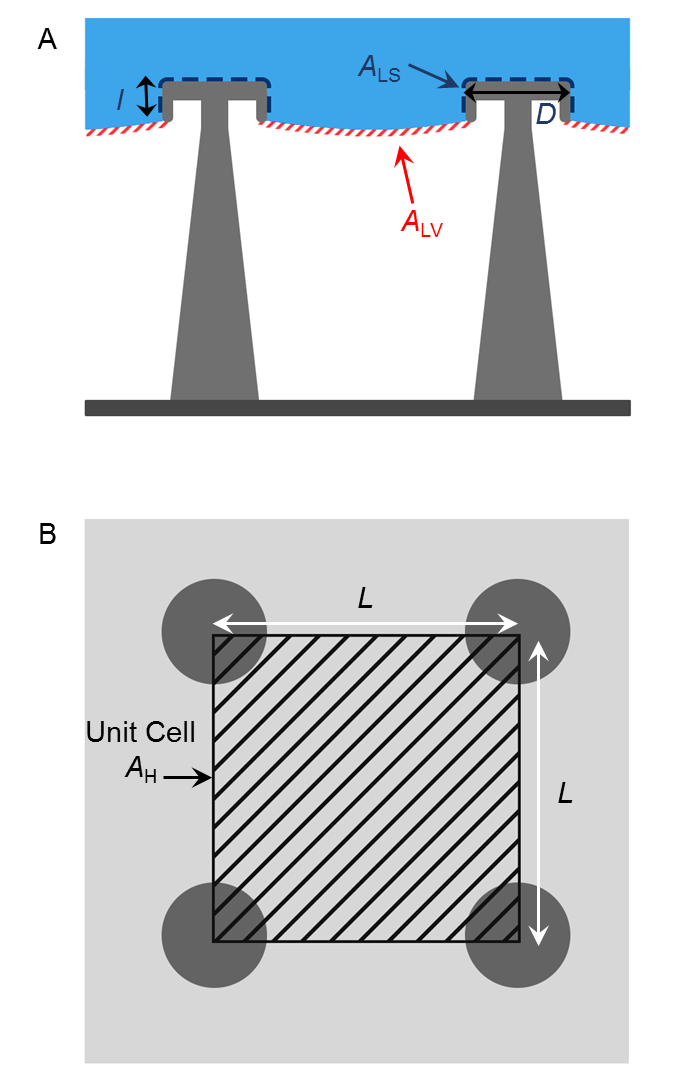


Figure S1: (A) Cross-section of DRP’s with a liquid meniscus stabilized at the doubly

reentrant edge. (B) A unit cell showing projected area for the calculation of $\phi_{\mathrm{LS}}$ and $\phi_{\mathrm{LV}}$.

*φ*_LS_ = *A*_LS_/*A*_H_ and *φ*_LV_ = *A*_LV_/*A*_H_, where *A*_LS_ and *A*_LV_ are the real liquid-solid and liquid vapor areas and *A*_H_ is the projected horizontal area. Figure S1 lists the various dimensions of the microtextures (*D*, *L*, and *l* refer to the diameter, pitch, and the height of the doubly reentrant feature of the DRPs).

(S1)

$$\phi_{\mathrm{LV}}=\frac{L^{2}-\frac{\pi D^{2}}{4}}{L^{2}}$$

(S2)

$$\phi_{\mathrm{LS}}=\frac{\frac{\pi D^{2}}{4}+\pi Dl}{L^{2}}$$

$\phi_{\mathrm{LS}}$ = 0.05 and $\phi_{\mathrm{LV}}$ = 0.97.

**Section S2**: Cassie-Baxter model

We used the Cassie Baxter model^1^ to predict the apparent contact angles on our microtextures

(S3)

$$\cos\theta_{r}=\phi_{\mathrm{LS}}\cos\theta_{0}-\phi_{\mathrm{LV}}$$

where $\theta_{r}$ is the apparent contact angle on the microtextured surface, $\theta_{0}$ is the actual (or intrinsic) contact angle on a flat and homogeneous surface (for terminology, see Ref.^2^), and *φ*_LS_ and *φ*_LV_ are liquid-solid and liquid-vapor area fractions, respectively, underneath the drop (Figure S1and Section S1).

**Table S2:** Predicted apparent contact angles using the Cassie-Baxter model

| ***D* (µm)** | ***L* (µm)** | ***l***  **(µm)** | $\boldsymbol{\phi}_{\mathbf{LV}}$ | $\boldsymbol{\phi}_{\mathbf{LS}}$ | **cos** $\boldsymbol{\theta}_{\mathbf{r}}$ | | | **Predicted** $\boldsymbol{\theta}_{\mathbf{r}}$ | | |
| --- | --- | --- | --- | --- | --- | --- | --- | --- | --- | --- |
|  |  |  |  |  | **Water** | **Hexadecane** | **Isopropanol** | **Water** | **Hexadecane** | **Isopropanol** |
| 20 | 100 | 2.5 | 0.969 | 0.047 | -0.954 | -0.923 | -0.923 | 162.6° | 157.3° | 157.3° |

**
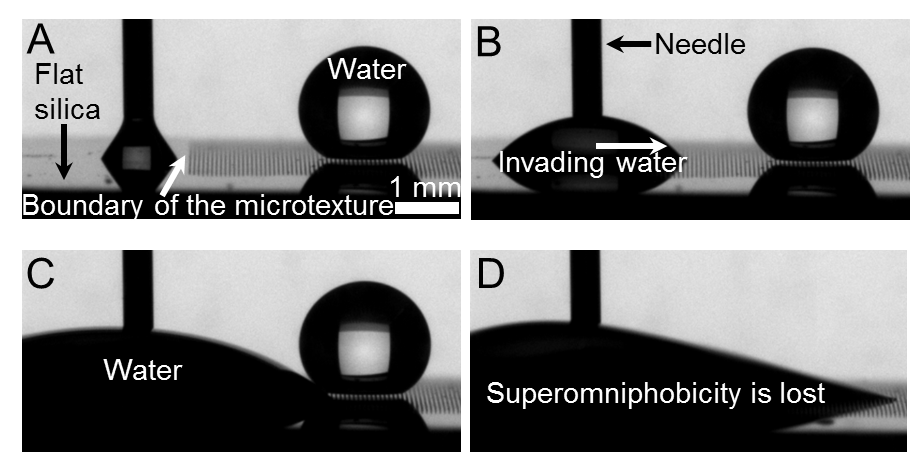
**

Figure S2: (A-D) Behavior of water advancing from the sides of DRPs microfabricated on a silica substrate. The invading liquid could access the pillar stem laterally and hence displaced the trapped air. Image reprinted from Ref.^3^ , Copyright (2019), with permission from Elsevier.


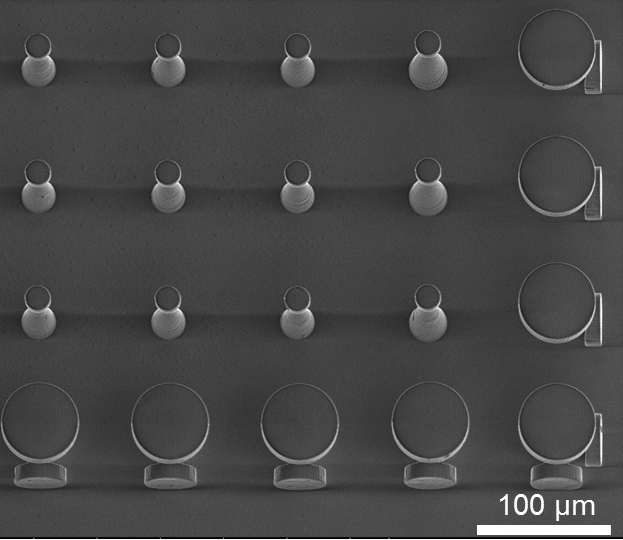


Figure S3: Scanning electron micrograph of arrays of GEMS design comprising an array of DRPs lined with DO-DRPs. Lateral caps on DO-DRPs were touching the floor.

**Section S3**: Confocal Microscopy

After immersing GEMS in wetting liquids, the solid-liquid-vapor interfaces were observed using a Zeiss LSM710 upright confocal laser scanning microscope. Trace amounts of fluorescent dyes, Rhodamine B (ACROS Organics) and Nile Red (ACROS Organics) (0.01 M), were used to visualize water and hexadecane, respectively. In a standard procedure, a sample was fixed on a petri dish using a double-sided tape and the probe liquid (comprising dye) was gently poured until the sample was completely covered by a *z* ≈ 5 mm column of solution. A 40X immersion objective was then lowered to the working distance and the experiments were immediately started. Sequential images (1024 px × 1024 px) were taken in the Z-stack mode. The intensity of the laser was 0.6 mW for water and 4 mW for hexadecane for optimum imaging. Subsequently, using the Imaris software from the Bitplane, we were able to create 3D-rendered surfaces and cross sections to visualize the interfaces.

**
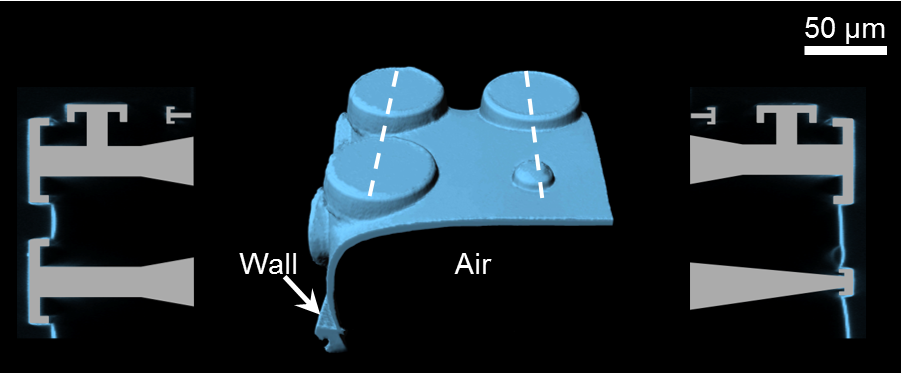
**

Figure S4: Computer-enhanced 3D reconstruction of GEMS-air-water interface after 10 min of immersion under a 5 mm-thick column of water. GEMS comprising an array of DRPs lined with DO-DRPs and a short wall with doubly reentrant profile. Cross-sectional views of the 3D reconstruction along the white dotted lines are shown on either side. The liquid menisci were stabilized at doubly reentrant edges.


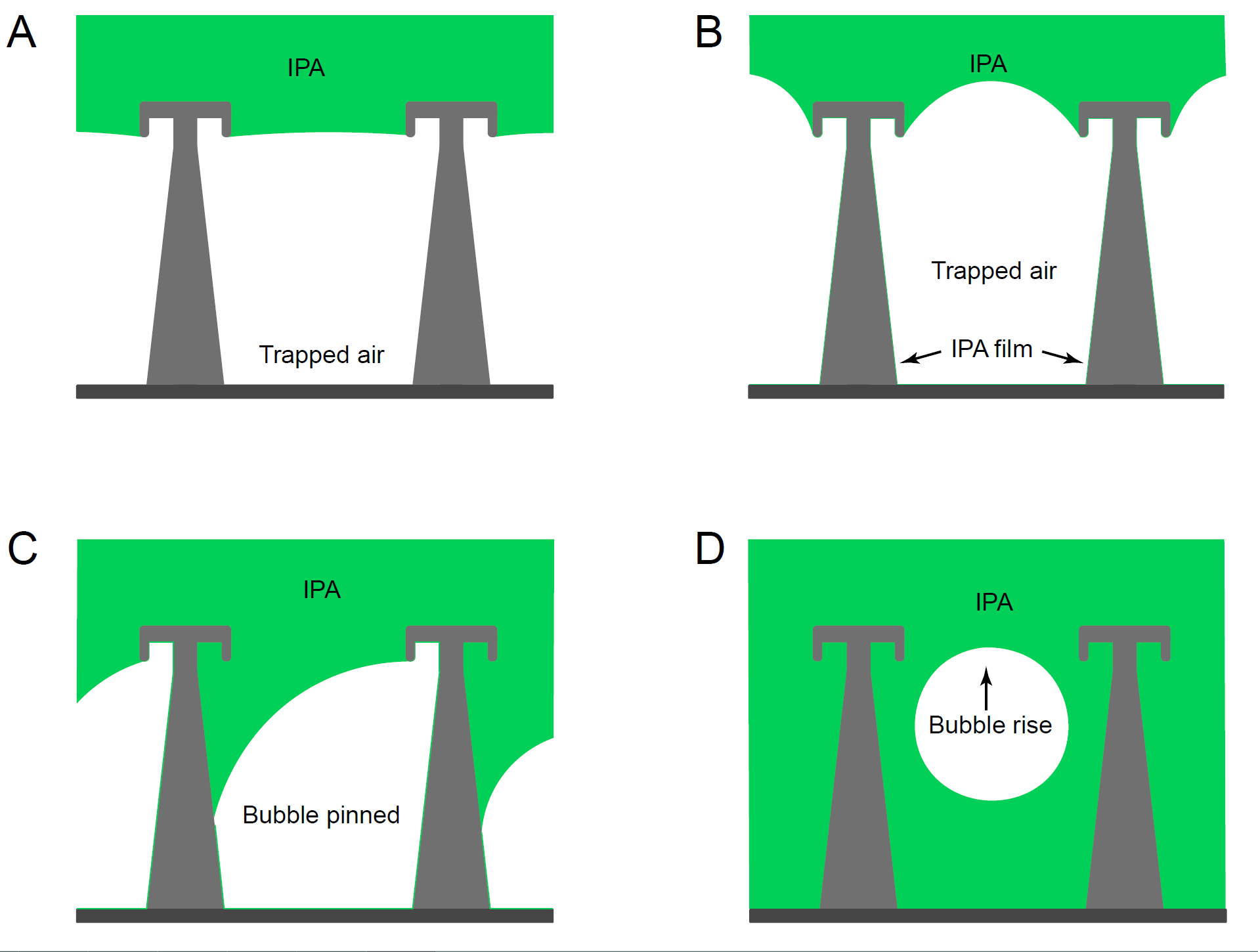


Figure S5: Schematic representation of immersion of GEMS in isopropanol. (A) Liquid meniscus is stabilized at doubly reentrant edge. (B) Formation of a thin condensed film of isopropanol on the pillar posts due to capillary condensation. (C) Isopropanol imbibes inside and meniscus failed asymmetrically. (D) The trapped air bubble rises upward due to buoyance and escapes the cavity due to negligible pinning force


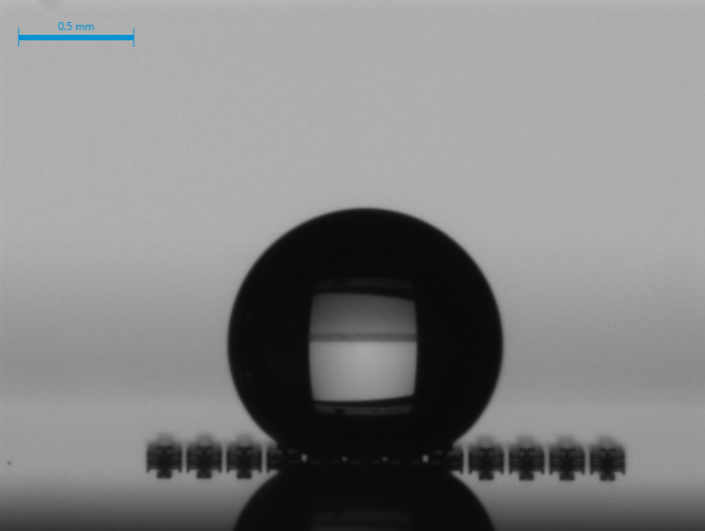


Figure S6: Droplet of IPA on GEMS – it does not penetrate into the microtexture.


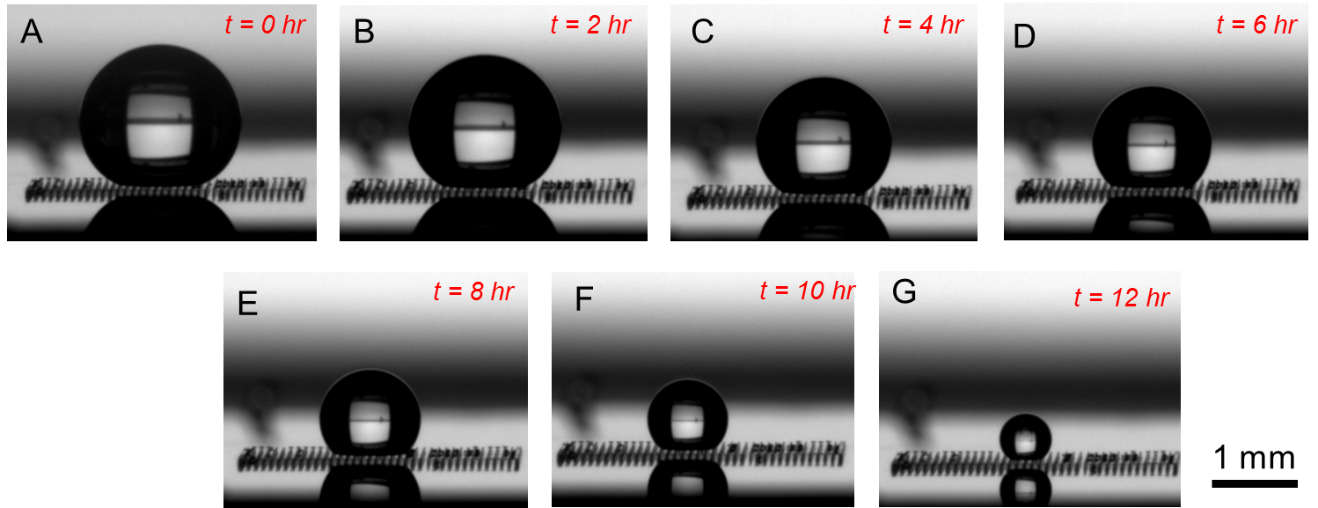


Figure S7: (A-G) Time evolution of a 6 µL water drop during its constant contact with GEMS, present under a high humidity environment (RH ≈ 92% ± 1%).

**Supplementary movies:**


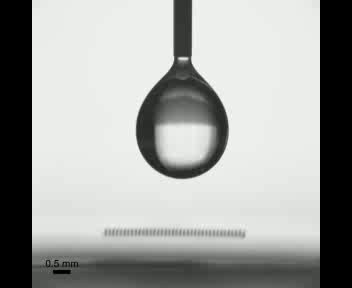


Movie S1: A water droplet impacting a surface with arrays of GEMS design comprising an array of DRPs lined with DO-DRPs. The movie was recorded at 10,000 fps.


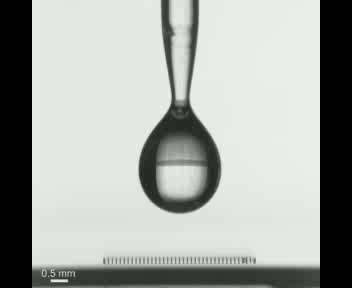


Movie S2: A hexadecane droplet impacting a surface with arrays of GEMS design comprising an array of DRPs lined with DO-DRPs. The movie was recorded at 10,000 fps.


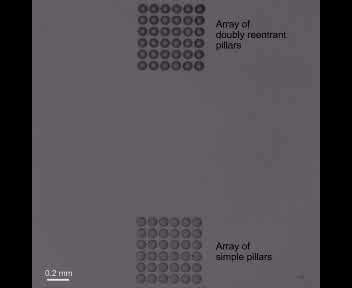


Movie S3: Arrays of simple and doubly reentrant pillars under immersion in water, both of them imbibed water spontaneously. The movie was recorded at 15 fps.


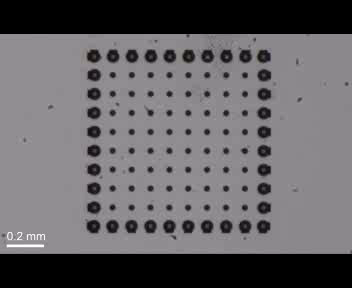


Movie S4: Surface with arrays of GEMS design comprising an array of DRPs lined with DO-DRPs under immersion in water, it trapped air under immersion. The movie was recorded at 15 fps.


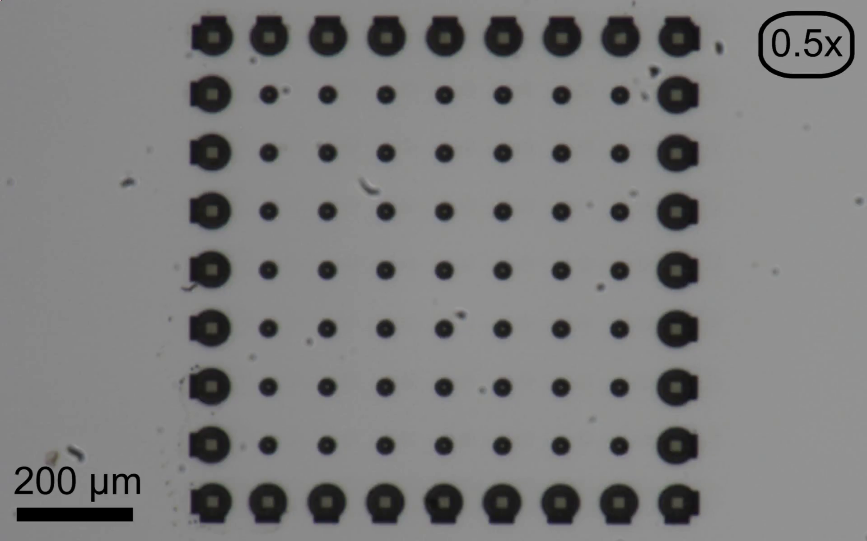


Movie S5: Surface with arrays of GEMS design comprising an array of DRPs lined with DO-DRPs under immersion in hexadecane, it spontaneously imbibed liquid under immersion. The movie was recorded at 15 fps.


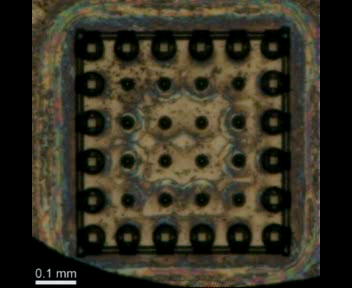


Movie S6: Surface with arrays of GEMS design comprising an array of DRPs lined with DO-DRPs and a short wall with doubly reentrant profile under immersion in hexadecane. It spontaneously trapped air; however, on small perturbations it lost the trapped air. The movie was recorded at 3000 fps.


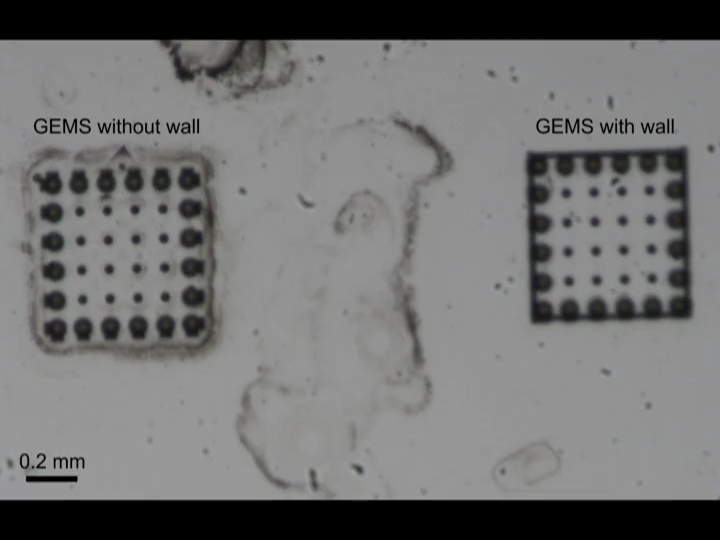


Movie S7: Surface with arrays of FDTS coated GEMS, comprising of an array of DRPs lined with DO-DRPs with and without the wall underneath under immersion in hexadecane, GEMS with the wall underneath trapped air under immersion. The movie was recorded at 163 fps.


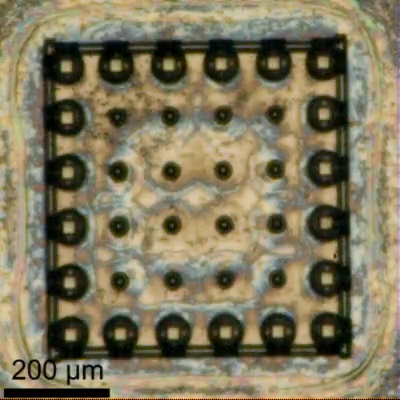


Movie S8: Surface with arrays of FDTS coated GEMS, design comprising an array of DRPs lined with DO-DRPs and a short wall with doubly reentrant profile under immersion in isopropanol. The movie was recorded at 3000 fps.

**References**

1 Cassie, A. B. D. & Baxter, S. Wettability of porous surfaces. *Transactions of the Faraday Society* **40**, 0546-0550, doi:DOI 10.1039/tf9444000546 (1944).

2 Abraham, M., Claudio, D. V., Stefano, S., Alidad, A. & W., D. J. Contact angles and wettability: towards common and accurate terminology. **5**, 3-8, doi:10.1680/jsuin.17.00002 (2017).

3 Arunachalam, S., Das, R., Nauruzbayeva, J., Domingues, E. M. & Mishra, H. Assessing omniphobicity by immersion. *Journal of Colloid and Interface Science* **534**, 156-162, doi:<https://doi.org/10.1016/j.jcis.2018.08.059> (2019).
